# The transient receptor potential channels in rheumatoid arthritis: Need to pay more attention

**DOI:** 10.3389/fimmu.2023.1127277

**Published:** 2023-03-01

**Authors:** Mengwen Niu, Feng Zhao, Rui Chen, Ping Li, Liqi Bi

**Affiliations:** ^1^ Department of Rheumatology and Immunology, China-Japan Union Hospital of Jilin University, Changchun, China; ^2^ Department of Cardiology, China-Japan Union Hospital of Jilin University, Changchun, China

**Keywords:** rheumatoid arthritis, transient receptor potential channels, inflammation, calcium signal, neuro-inflammation, inflammatory pain

## Abstract

Rheumatoid arthritis (RA) is characterized by the augment of vascular permeability, increased inflammatory cells infiltration, dysregulated immune cells activation, pannus formation and unbearable pain hyperalgesia. Ca^2+^ affect almost every aspect of cellular functions, involving cell migration, signal transduction, proliferation, and apoptosis. Transient receptor potential channels (TRPs) as a type of non-selective permeable cation channels, can regulate Ca^2+^ entry and intracellular Ca^2+^ signal in cells including immune cells and neurons. Researches have demonstrated that TRPs in the mechanisms of inflammatory diseases have achieved rapid progress, while the roles of TRPs in RA pathogenesis and pain hyperalgesia are still not well understood. To solve this problem, this review presents the evidence of TRPs on vascular endothelial cells in joint swelling, neutrophils activation and their trans-endothelial migration, as well as their bridging role in the reactive oxygen species/TRPs/Ca^2+^/peptidyl arginine deiminases networks in accelerating citrullinated proteins formation. It also points out the distinct functions of TRPs subfamilies expressed in the nervous systems of joints in cold hyperalgesia and neuro-inflammation mutually influenced inflammatory pain in RA. Thus, more attention could be paid on the impact of TRPs in RA and TRPs are useful in researches on the molecular mechanisms of anti-inflammation and analgesic therapeutic strategies.

## 1 Introduction

Rheumatoid arthritis (RA) is a systemic inflammatory autoimmune disease characterized by the infiltration of T cells, B cells, and inflammatory cells such as neutrophils, macrophages, and so on within joints. Inflammatory factors and autoantibodies produced and secreted by immune cells act on cartilage, tendons, ligaments, and bone tissues, causing joint swelling, pain hyperalgesia, pannus formation, and eventually bone destruction (1, 2). It has a high prevalence and disability rate, bringing a huge economic burden and pressure to society and families (3). While the pathogenesis of RA remains unclear, so, it is still a long way to go.

Ca^2+^ affect almost every aspect of cellular functions, involving cell proliferation, apoptosis, migration as well as signal transduction. Calcium ion as a necessary molecule of physiological signal transduction in eukaryotes, it functioned as a central manipulator of inflammation as well as immune response in RA (4). As non-selective Ca^2+^ permeable channels, TRPs expressed on neutrophils (5, 6), chondrocytes (7, 8), fibroblast-like cells (FLS) (9) and nervous system (10, 11) have been proven to regulate cation flow and potential changes, helping them participate in RA.

The TRP channel superfamily consist of 28 members (27 in humans) in mammals (3). Based on sequence homology, the superfamily is divided into seven subfamilies in mammals (12). Four subfamilies of TRPs have been well studied which are known as TRPCs, TRPVs, TRPMs, and TRPA1. There are also different categories within each subtype, including the Canonical subfamily (TRPC1-7), the Vanilloid subfamily (TRPV1-6), the Melastatin subfamily (TRPM1-8) (13, 14). As they can be activated by diverse stimuli no matter in extracellular and intracellular circumstances, for example the alteration of the temperature, osmolarity, depletion of calcium stores, as well as cytokines. Therefore, they play a series of important roles in physiological and pathological status (15–17).

Though TRPs have been studied in many other diseases such as cardiovascular, renal, tumor, lung inflammation and other inflammatory diseases, researches on their roles in rheumatoid arthritis are limited. We carefully selected representative articles and explore the different and indispensable roles of these TRPs in RA pathogenesis and in cold and inflammatory stimuli perceptions as well as their influence on neuro-inflammatory interaction outcomes. At the same time, we also show our ideas as we illustrate the relevant issues.

## 2 Role of TRPs in vascular permeability

RA patients typically complain of swelling and stiffness of joints. Vascular endothelial cells (ECs) are involved in immune and inflammatory responses because they can produce different cytokines and react with immune-inflammatory factors, causing the augmentation of endothelial permeability (18). The increase of Ca^2+^ in ECs closely related to endothelial cell permeability (19). However, the exact molecular mechanism involved in the increase of endothelial permeability is not yet completely known.

### 2.1 TRPs promote the Ca^2+^ influx of ECs

The inner layer of blood vessels consists of adjacent ECs and their tight connections. The integrity of the endothelial layer was guaranteed by intercellular linkages comprising of adherents and tight junctions (20, 21). The calcium influxes/transients cause adherents junction disassembly, cytoskeletal rearrangements and facilitate ECs retraction. Therefore, the morphology of ECs was transformed to a round shape, and gaps between them were augmented, inducing their permeability increase (21, 22). The concentration of intracellular calcium ion promoted with the increased opening of TRPC1 on ECs, which accelerated the formation of actin stress fibers and enhanced the stretching and deforming ability of ECs, thus leading to increased permeability (19). The TRPC6 expressed in ECs collaborated with platelet/endothelial cell adhesion molecule-1 (PECAM) to surround leukocytes during their trans-endothelial migration (TEM) and could help them in their migration. The down-regulated expression of TRPC6 or shRNA knockdown in ECs arrested neutrophils over the junction, similar to when PECAM was blocked (23).

**Figure 1:**
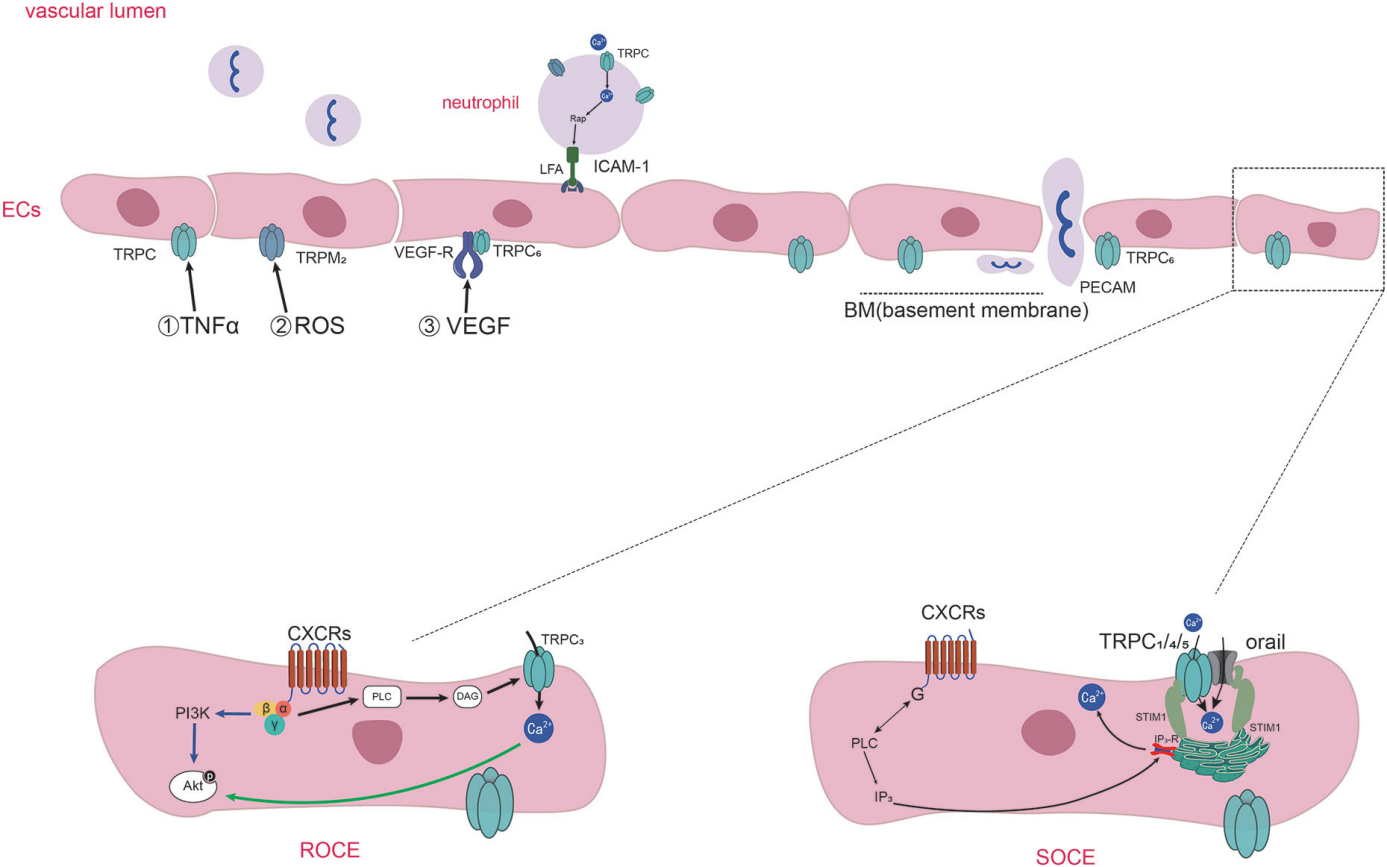
Diagram of TRPs-mediated Ca^2+^ entering into endothelial cells and neutrophils chemotaxis and adhesion in inflammation. Diagram of TRPs-mediated Ca^2+^ entering into endothelial cells and neutrophils chemotaxis and adhesion in inflammation. Inflammatory stimuli (such as TNF-α, ROS, VEGF) influence the expression and activation of TRPs expressed on ECs. Activation of specific GPCRs on the cell surface causes the activation of PLC which cleaves the membrane phospholipid PIP2 into inositol 1,4,5-trisphosphate (IP3) and diacylglycerol (DAG). DAG induce the activation of TRPC3, TRPC6, and TRPC7 channels, and the way of the influx of Ca^2+^ called receptor operated Ca^2+^ entry (ROCE). IP3 binds to IP3 receptors (IP3Rs) present on the ER, causing Ca^2+^ release from intracellular stores. Then the Ca^2+^ sensor stromal interaction molecule1(STIM1) proteins gather to the membrane of ER and activate the channels of orai1 and TRPC1/4/5, thus promoting extracellular Ca^2+^ influx which is called as SOCE. TRPCs also mediate the adhesion of neutrophils through regulating the anchor link of neutrophil and epithelial cells. TRPC6 expressed in endothelial cells colocalize with PECAM, surrounding leukocytes and promoting neutrophil trans-endothelial migration.

Among the 28 identified and widespread mammalian TRP channel isoforms, at least 19 are expressed in ECs (24, 25), and the transient receptor potential canonical channels (TRPCs) are the subfamily of TRPs closely related to endothelial cell permeability. ECs express six non-selectively cation permeable TRPCs, which are TRPC1, TRPC3, TRPC4, TRPC5, TRPC6, and TRPC7 (25, 26). Most studies agreed that the influx of Ca^2+^ into ECs through TRPC1, TRPC4, and TRPC5 was mediated by the store-operated Ca^2+^ entry (SOCE), starting with the depletion of Ca^2+^ in endoplasmic reticulum (ER) stores (27, 28). But, some studies had questioned whether the TRPC1/4/5 channels mediated the increase of Ca^2+^ through SOCE (29). It has been reached an agreement that the influx of Ca^2+^ through TRPC3, TRPC6 and TRPC7 was induced by the receptor-operated Ca^2+^ entry (ROCE). An important stimulant of TRPCs is the α-subunits of G-proteins. Thus, inflammatory stimuli cause the activation of G-protein-coupled receptors (GPCR) and the phospholipase C (PLC) promote the opening of TRPC channels (30, 31). Even though TRPCs may also activated by some other stimulates, the mainly same effect is the increase of calcium influx in ECs. The mechanisms of different TRPC subfamilies in mediating calcium ions entering into endothelial cells are shown in **Figure 1**.

### 2.2 The activation of TRPs by inflammatory factors

Inflammatory factors interact with TRPs on vascular ECs of the inflammatory sites, leading to increased vascular permeability. TNF-α works as a major inflammatory factor in RA, it is often used as an inflammatory mediator to simulate the inflammatory reaction process of RA (32–35). TNF-α could cause the TRPC1 expression which medicated increased Ca^2+^ entry through SOCE, representing an important mechanism of endothelial injury through TRPC1 (19, 36). Ca^2+^ flowing through TRPC1 can also increase TRPC1 expression through positive feedback. Protease-activated receptor-1(PAR-1) activation elicited cellular responses including NF-κB activation and increased Ca^2+^ influx through TRPC1. Ca^2+^ then regulated the expression of TRPC1 expression which may be a vicious circle resulting in vascular permeability (36–38). Besides, inflammatory mediators, such as thrombin were reported to be increased in RA (39, 40). Thrombin induced the phosphorylation of TRPC1 by PKCα activation, resulting in Ca^2+^ entry and the increase in permeability in confluent endothelial monolayers (41). The inflammatory factor thrombin could also promote the association between TRPC6 and phosphatase tensin homologue (PTEN) which worked as a role of the scaffold for TRPC6, enabling the expression of TRPC6 on cell surface, which made the increase of Ca^2+^ entry (42). Therefore, the increased Ca^2+^ entry through TRPC1/6 plays an important role in augmenting endothelial permeability in RA.

Reactive oxygen species (ROS) are cellular metabolites that contain at least one oxygen atom and one or more unpaired electrons (43). ROS could damage DNA, proteins, lipids, and many other molecules (44). Phagocytes and recruited immune cells could produce a lot of ROS in the RA synovitis microenvironment (45–47). Increased ROS caused by Oxidative stress can destroy endothelial integrity by leading to Ca^2+^ influx into ECs, and disrupt the tight junctions between them (48). TRPM2 expressed in ECs can be activated by ROS and play a role as Ca^2+^-permeable channel. The sub-lytic concentration of H_2_O_2_ induced an increase in ECs intracellular Ca^2+^ by stimulating Ca^2+^ entry through the TRPM2, and subsequent reduction of transendothelial resistance (49).

VEGF was first isolated and named vascular permeability factor (VPF), a potent vascular permeability-enhancing cytokine and a selective mitogen for endothelial cells (50). VEGF expression was increased in vascular endothelium and synovial tissue in RA joints and was associated with inflammation and angiogenesis (51–54). VEGF could also provoke ROS production with the participation of NOX, followed by the autophosphorylation of VEGFR2 (55). Corroborating with the above paragraph, VEGF and ROS can act as two interactive inflammatory molecules synthesized at the site of inflammation, and weaken the endothelial functions. Decrease expression of TRPC6 in human microvascular endothelial cells (HMVECs) restrained the VEGF-mediated increases in Ca^2+^ and migration of HMVECs (56). Therefore, TRPC6 is an indispensable cation channels needed in the VEGF-mediated increase of Ca^2+^ in HMVECs.

VEGFs can identify and combine with their cognate VEGF receptors, including VEGFR1, VEGFR2, and VEGFR3, as well as some co-receptors (57). VEGF medicated endothelial hyperpermeability in a phospholipase C (PLC)-IP3 pathway, which result in extracellular Ca^2+^ entry *via* the plasmalemma store-operated TRPC1. VEGF augmented the interaction of IP3R with TRPC1 (58). VEGF could activate a receptor-operated cation current in HMVECs, in which VEGF bound with VEGFR2 which activated heterologously co-expressed TRPC3/6 channels, such as the style of VEGFR2-TRPC3 and VEGFR2-TRPC6 on cells (59). In conclusion, we hypothesized that when VEGF binds to the corresponding VEGFR or co-receptors on ECs, it may cause the activation of TRPC channels in the form of linking the body with these receptors, which leads to the increase of intracellular calcium ions and the increase of endothelial cell permeability.

## 3 Role of TRPs in neutrophils’ TEM and proteins citrullination

Neutrophils are the main force of the first line of immune defense and always be recruited to sites of inflammation as soon as possible. The recruitment of neutrophils and their activation against pathogen-associated molecular patterns (PAMPs) or damage-associated molecular patterns (DAMPs) are highly coordinated and tightly regulated processes that involve the activation of various kinds of receptors as well as many ion channels (60, 61). Fluctuations in intracellular Ca^2+^ levels are hallmarks in the above complex processes of neutrophils. Hence, TRPs, as non-selective permeable Ca^2+^ channels distributed on neutrophil, have an important role in the functions of neutrophil in RA.

### 3.1 The contribution of TRPs to neutrophils TEM

Leukocyte TEM is the first step of their participating in the immune response and is vital to inflammation (62–66). Neutrophils can be activated by plentiful of “neutrophil-active” chemo-attractants, which initiate the TEM process, including different kinds of chemokines and cytokines (such as CXCLs) as well as fragments of complement (65). TRPC6(-/-) neutrophils had decreased Ca^2+^ transient during initial adhesion, resulting in reduced activation of Rap1 and β2 integrins and reduced binding to ICAM-1. This suggests that the TRPC6 on neutrophils is a key functional channel protein in CXCL1’s recruitment of inflammatory cells from the bloodstream (67). The deletion of TRPC1 led to reduced migration and chemotaxis of neutrophils due to disruption of Ca^2+^ gradient homeostasis within TRPC1-/- neutrophils, resulting in more disordered migration and reduced directed migration of neutrophils (68).

A large number of activated neutrophils were found in both peripheral blood and synovial tissue from RA patients (69). Different kinds of chemokines are manifested by studies highly expressed in the serum and synovial fluid in RA, for instance, the CXC-chemokines, the CC-chemokines, and the CX3C-chemokine CX3CL1 (70–73). Intracellular Ca^2+^ flow from the extracellular space induced by CXCR2 as a chemotactic agent is affected in TRPC6(-/-) neutrophils. Deletion of TRPC6 inhibited phosphorylation of AKT and MAPK molecules, which were downstream of CXCR2 receptors, attenuated actin remodeling, and thus impeded the chemotaxis of neutrophils (74). TRPC6 could also be activated by the CXC-type G-protein-coupled chemokine receptors upon stimulation with macrophage inflammatory protein-2 (MIP-2), which was essential for the arrangement of filamentous actin of migrating neutrophils (75).

TRPM7 is a member of the subfamily TRPMs and works as not only an ion channel but also kinase activity. If the TRPM7 channel or kinase activity was blocked, the ability of human neutrophils to migrate along CXCL8 chemokine gradients was significantly reduced. Kinase activity of TRPM7 affected the Akt1/mTOR signaling pathway and regulated neutrophil migration and function (5). Recent researches showed that the expression of CD147 on neutrophils from RA patients was higher than that in healthy group (76). TRPM-7-mediated the increase of Ca^2+^ and induced chemotaxis, adhesion ability, and invasiveness of RA neutrophils response to CD147. When treated with the transient receptor potential melastatin 7 small interfering RNA(si-TRPM7), the chemotaxis and the relative adhesion rates of neutrophils were reduced significantly (6).

### 3.2 Role of TRPs in the formation of citrullination proteins

Once neutrophils are activated by inflammatory stimuli, their intracellular Ca^2+^ concentration will be enhanced. The calcium-dependent peptidyl arginine deiminases (PADs) will be stimulated by the increased concentration of Ca^2+^ in neutrophil. PADs convert the protein arginine residues to citrulline residues, this process is called protein citrullination (77). The autoantibodies that recognize citrulline protein antigen (ACPAs) may have been present prior to diagnosis, and studies have shown that it is a major pathogenic molecule of RA (78, 79). In the process of citrullination, the PADs are the key enzymes, therefore, it is necessary to explore the influencing factors of PADs activity. So, we consider that TRPs that can cause calcium influx may play a role in promoting the activity of PADs in neutrophil.

#### 3.2.1 TRPCs and the activation of PADs

It has been manifested that Calcium is the primary factor in the activation of PADs enzymes, because it can motivate structural changes of PADs. PADs have five calcium-binding sites, and the enzyme active sites will be exposed when they bind to calcium ions (80). At the same time, the conformational changes are accompanied by the transition from inactive to active PADs.

**Figure 2:**
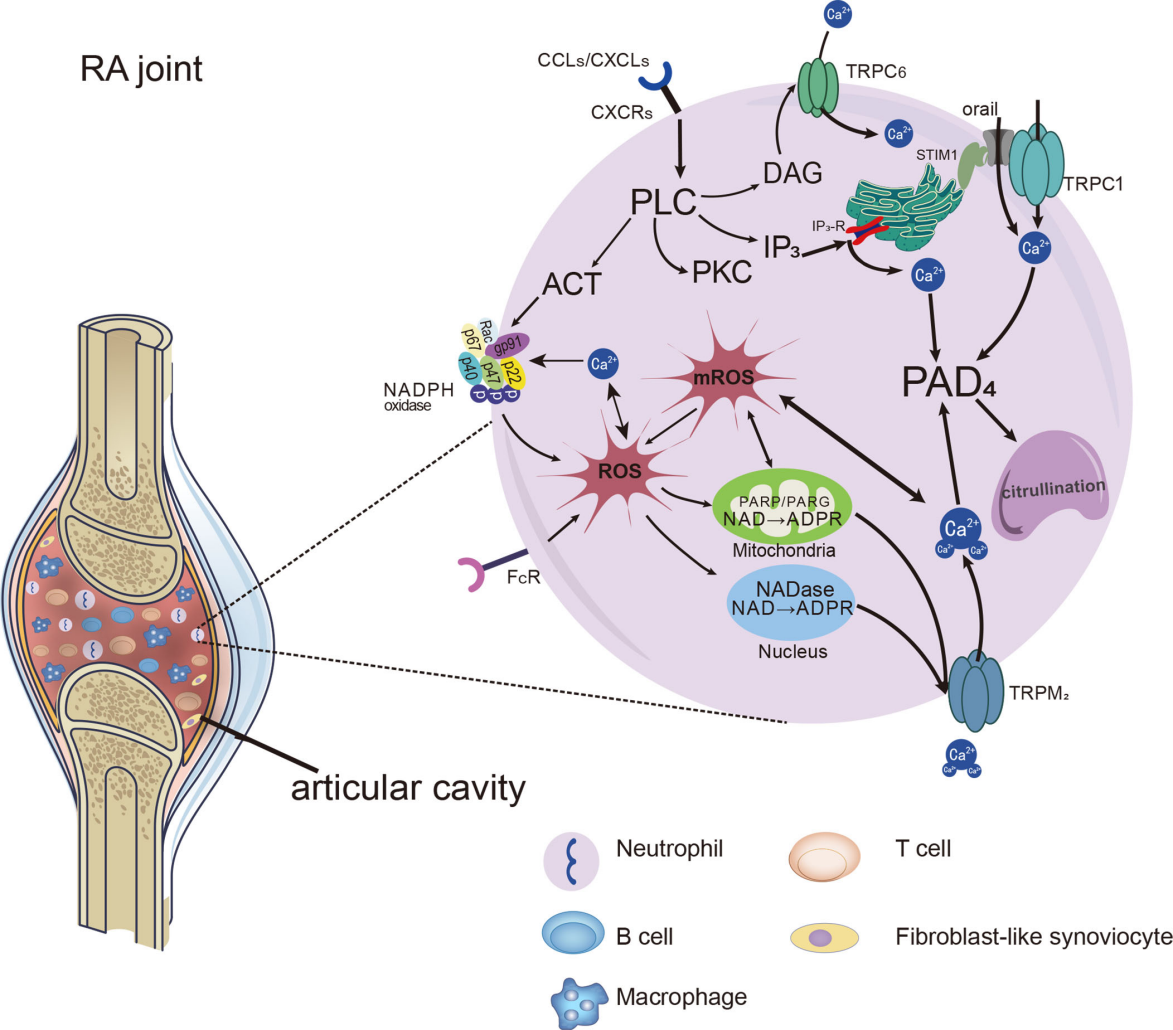
Distinct Ca^2+^ signals and downstream cellular events associated with TRPs in neutrophils. Distinct Ca^2+^ signals and downstream cellular events associated with TRPs on neutrophils in RA joints. Chemokines and immune-inflammation factors stimulate the activation of receptors on neutrophils, leading to the activation of PLC and the production of molecules downstream of it. NADPH oxidase can be activated and make an increase in ROS production. ROS leads to the increase of ADPR production, which acts as the activator of the TRPM2 channel and further promotes the entry of Ca^2+^ into cells. In addition, DAG, as the activator of TRPC6, can induce increased calcium influx through ROCE. With the addition of Ca^2+^ entering by SOCE, cytosolic Ca^2+^ level rapidly increased by the SOCE, ROCE and ROS/TRPs mechanisms. Ca^2+^ catalyzes PADs which facilitate citrullination, and even promote the formation of NETs in neutrophils.

It has been shown that three subtypes of TRPs are expressed in human and mouse PMNs, including TRPCs, TRPMs and TRPVs (60). Receptors on neutrophils can be activated by inflammatory stimuli, leading to the activation of PLC which cleaves the membrane phospholipid PIP_2_ into inositol 1,4,5-trisphosphate (IP_3_) and DAG. DAG induce the activation of TRPC6 channels, through which the Ca^2+^ entry into neutrophils (81). IP_3_ binds to IP_3_ receptors (IP_3_Rs) present on the ER, causing Ca^2+^ release from intracellular stores. Then the Ca^2+^ sensor stromal interaction molecule 1**(**STIM1) proteins gather to the membrane of ER and activate the Orai1 and TRPC1, thus promoting extracellular Ca^2+^ influx neutrophils (82). Increased intracellular Ca^2+^ concentration in neutrophils promotes PADs activation, which in turn leads to the formation of citrullination proteins or neutrophil extracellular traps (NETs). We have shown evidence that TRPCs offer assistance in Ca^2+^ influx through SOCE and ROCE during the process of the recruitment and activation of neutrophils, and summarized in **Figures 1**, **2**.

#### 3.2.2 TRPM2 and the activation of PADs

Interestingly, most of the stimuli that lead to an increase in the calcium concentration of neutrophils also lead to the production of ROS. ROS generation and Ca^2+^ concentration in synovial neutrophils from RA patients were increased, and proinflammatory cytokines such as GM-CSF and TNF-α enhanced ROS production in neutrophils by activating NADPH oxidase (83). Moreover, ROS-dependent NETs formation was associated with enhanced PADs activity (84, 85). In line with those described above, RA neutrophils presented increased formation of NETs with elevated ROS and Ca^2+^ concentrations, accompanied by enhanced PADs mediating citrullination (86). It seems apparent that ROS and Ca^2+^ play a mutual interaction in promoting PADs enzyme activity.

Notably, TRPM2 cationic channels play a mediating role in regulating Ca^2+^ mobilization and oxidative stress in neutrophils. They regulate calcium influx in a way that is different from the SOCE and ROCE pathways mentioned above. The activating ligand for TRPM2 is ADP-ribose (ADPR), which is increased when intracellular ROS levels are high and works with intracellular calcium to activate TRPM2 (87–89). H_2_O_2_ was shown to prompt ADPR generation, which acted as an activator of TRPM2. The increased calcium through TRPM2 could further induce production of the ROS (90). FcγRIIIb activated by immune complexes evoked protein kinase C (PKC) activation, which promoted NADPH-oxidase in ROS production, leading to activation of TRPM2 channels, allowing significant up-regulation of extracellular Ca^2+^ into the neutrophil (91). Compared with the control group, the concentration of intracellular free Ca^2+^ and ROS production in neutrophils were significantly increased in patients with RA. The intracellular Ca^2+^ concentrations were decreased by anti-TRPM2 therapy (92). Hence, we consider that the TRPM2 work as a bridge of inflammation or oxidative stress and calcium signal in the NADPH oxidase/ROS/TRPs/Ca^2+^/PADs pathway, as shown in **Figure 2**.

## 4 Role of TRPs in pannus formation

Angiogenesis/neovascularization is an invasive event in the pathogenesis of RA which is characterized by synovial tissue proliferation. Osteochondral angiogenesis of RA is characterized by fibrovascular tissue expressing VEGF and increased proliferation of ECs and fibroblast-like synovial cells (FLS) (93). VEGF, a key governor of angiogenesis, can regulate angiogenesis *via* VEGFR2 (94). The overexpression of TRPV4 up-regulated VEGFR2 trafficking and activation, controlling ECs migration and angiogenesis (95). It was also found to induce endothelial progenitor cells (EPC) proliferation and migration, which facilitated angiogenesis and enabled the development of RA (96). TRPC3, TRPC4, and TRPC5 were essential for the formation of tubular structures and revealed an essential contribution of TRPCs to the vitro tubulogenesis of endothelial cell lines (97). TRPC6 was also a cationic channel necessary for the related pathway processes of VEGF-mediated cytoplasmic calcium increase and angiogenesis (56, 98).

Accumulating data has proved that the apoptosis rate of FLS in RA is decreased, and their proliferative and invasive properties lead to progressive destruction of cartilage and bone (99, 100). TRPC6 was found somewhat higher in RA-FLS than that in OA-FLS. TRPC6 deficiency in mice weakened the development of experimental RA and CIA models and inhibited FLS invasiveness and proliferation (101). The expression of TRPM7 was significantly increased in RA-FLS, TRPM7 may have a role in reduced FLS apoptosis because suppression of TRPM7 channels increased RA FLSs apoptosis *in vitro*, and this was associated with endoplasmic reticulum (ER) stress activation (102). Another study found that TRPA1 activation was associated with decreased proliferation of FLS, an effect that was substantially enhanced by TNF preincubation. The reason may be that activation of TRPA1 increased calcium flux and substantially reduced cell viability by inducing necrosis (103). As we can only retrieve a few studies on the correlation between TRPs and FLS proliferation or apoptosis at present, the assured roles of TRPs in promoting the proliferation or apoptosis of the FLS are not yet clear. We are looking forward to detailed studies in this field.

## 5 Role of TRPs in pain perception and neuro-immune communication

RA is a serious and persistent painful disease of the distal joints in which neurogenic components are supposed to participate in its pathology. Pain is the most common complaint of patients in the rheumatology department, and the threshold of patients for pain is often lower than that of healthy people, that is, pain hyperalgesia (104). There are a large number of sensory nerve fibers in the synovium of the joint. Located on these sensory nerve fibers, TRPs act as an important superfamily of proteins that mediate heat and mechanical pain. Recent findings regarding the role of TRPs in cold and inflammatory pain hypersensitivity spouted. The mechanisms of TRPs in temperature perception, nociception, sensitization of inflammatory stimuli, and neuro-immune communication will be stated in detail in the following sections.

### 5.1 Role of TRPs in cold allodynia in RA

When the weather gets cold, there are many cases where patients with RA have to visit the clinic because of unbearable joint pain. Although the mechanism of the effect of cold environment on arthritis patients is not clear, it has been shown to be one of the common precursors and aggravating factors of arthritis.

#### 5.1.1 TRPA1 in the perception of cold and blood flow sustainment

TRPA1 on neurons can perceive the changes of thermal (< 17°C), chemical (menthol, formalin, reactive lipids, etc.), and mechanical (sting, pinch, etc.) (105). Studies established that TRPA1 acted as a cold sensor in experimental models, revealing that TRPA1 was a major sensor for noxious cold (106, 107). TRPA1 protein and mRNA were overexpressed in both peripheral and central nervous system of CFA treated mice, and TRPA1 played a vital role in the development and maintenance of cold pain hyperalgesia. However, pretreatment with TRPA1 antagonist HC-030031 inhibited the above results (108). While, as a opposite, one study argued that WT and TRPA1 KO mice had no difference in the perception of thermal pain in the inflamed claw compared with that of naive mice (109). Additionally, another study hinted that TRPA1 agonists can sensitize the noxious reflex withdrawal to heat, but not cold (110). Why do they have contradictory conclusions? The reasons may be the ways and degrees of cold stimuli and the detection methods they used, and the test time points after stimulation they set. Therefore, different investigational methods may reflect controversial results.

In one study, cold exposed CFA arthritis mice developed bilateral pain sensitivity in their knee joints, which was dependent on the cold sensor, TRPA1. Moreover, blood flow was seen to increase in the joint after cold exposure which also depended on TRPA1, and due to the vasodilatory neuropeptides, including Substance P (SP) and calcitonin gene-related peptide (CGRP) (111). Thus, TRPA1 may act to influence not only its established nociceptive actions within the joint but also blood flow *via* neuropeptides. Then, why TRPA1 can medicate the pain hyperalgesia and still cause increased blood flow than that of the control group after cold exposure? When the animals were at room temperature, blood flow of CFA-treated joints was lower than that in control joints. However, under the stimulation of a cold environment, the sympathetic nerves of the CFA group were excited and secreted various vasodilatory substances. SP and CGRP participated in the stabilization of blood flow in the synovium after cold stimulation (111). Similarly, blood vessel constriction in the inflammation site was most obvious within 0 - 2 minutes after cold stimulation, and then gradually returned to the baseline level. The initial cold-induced vasoconstriction was mediated *via* TRPA1-dependent superoxide production, and the subsequent restorative blood flow component was also dependent on TRPA1 activation being mediated by sensory nerve-derived dilator neuropeptides CGRP and SP, and also NOS-derived NO (112, 113). Therefore, blood vessels in the inflamed joint will have a process of the first contraction and then relaxation after being cold, and different experimental detection points will have discrepant results. Increased blood flow is due to the effects of TRPA1 and vasodilatory substances, which also cause hypersensitivity to pain.

#### 5.1.2 TRPM8 in the perception of cold and collaboration with TRPA1

TRPM8 is a non-selective cationic channel that can be activated by low temperatures and cooling agents, and plays a role in the perception of cold environments. The response of sensory neurons and intact sensory nerve fibers in TRPM8-deficient mice to cold was significantly weakened *in vitro* (114, 115). Cold pain associated with CFA-induced inflammation was significantly attenuated in TRPM8(-/-) mice (116). However, one study showed that TRPA1 mediated cold pain in CFA-induced arthritis, but TRPM8 had no effect. The expression of TRPA1, but not TRPM8, increased in CFA-induced inflammation, and knockdown of the TRPA1 gene prevented and reversed inflammation-induced cold hyperalgesia (117). A few experiments found that the threshold of TRPM8 activation was 25°C, consistent with the pleasant/cool feeling induced by menthol products and so, had no effect on cold pain. Different from the above view, TRPM8 and TRPA1 had synergistic effects on cold nociceptive perception (118). A group of researchers found that CFA-induced inflammatory cold pain was selectively mediated by glial cell line derived neurotrophic factor family receptors (GFRα3), which were expressed in a subpopulation of TRPM8 sensory neurons that act as cold nociceptors (119, 120). They then provided new insight that the neurogenic inflammation upstream of the GFRα3 and TRPM8 promoted TRPA1’s perception of cold pain, which was in a TRPM8-dependent manner (121). According to the above, TRPM8 joins hands with TRPA1 and serve an important role in cold pain in CFA-induced arthritis.

### 5.2 TRPs in inflammatory pain (heat and mechanical)

Thermal (heat) and mechanical noxious stimuli are detected by specialized nerve endings, which transform the stimuli into electrical signals and convey the stimuli to the central nervous system, causing thermal and mechanical pain. The basis for hyperalgesia is the sensitization of the nociceptive system to stimuli (hypersensitivity), moreover, the threshold for the excitation of nociceptive neurons is lowered(low threshold), and therefore, the responses to noxious stimuli are heightened (122). RA patients suffer from hypersensitivity to heat and mechanical joint pain caused by inflammatory factors and joint movement. In the following sections, we emphasize the introduction of inflammatory thermal (heat) and mechanical pain, mainly on the mechanisms involved in TRPV1 and TRPA1.

#### 5.2.1 Role of TRPV1 in inflammatory pain

The TRPV1 is expressed on sensory neurons and can sense changes of heat (temperature >43°C), proinflammatory substances, lipoxygenase products, endocannabinoids, protons, and so on (123). TRPV1 is involved in two typical inflammatory hyperalgesia: thermal and mechanical pain. Peripheral distribution of TRPV1 played a major role in CFA-induced thermal hyperalgesia, but to a lesser extent in mechanical hyperalgesia. While, spinal cord distribution of TRPV1 act as an important role in the perception of both types of hyperalgesia (124).

There was a significant interaction between the inflammatory state and TRPV1 activation, resulting in inflammatory pain perception (125). Firstly, TRPV1 receptor protein was upregulated during CFA-induced inflammation, and mediated inflammatory hyperalgesia (126–128). Secondly, the activation of TRPV1 is regulated by its phosphorylation status which is dependent on the balanced actions of protein kinases and protein phosphatases (129). The activity of protein kinases and protein phosphatases are often activated or inhibited in inflammatory circumstances. In CFA-induced arthritis models, increased expression of phosphorylated protein kinase A phosphorylated TRPV1 and sensitized it, resulting in inflammatory mechanical and thermal pain (130). Furthermore, evidence showed that TRPV1 phosphorylation by Cdk5(Cyclin-dependent kinase 5, an important serine/threonine kinase) promoted the distribution of TRPV1 in the plasma membrane and contributed to thermal hyperalgesia during inflammation post-CFA (131).

#### 5.2.2 Role of TRPA1 in inflammatory pain

At the beginning of this section, we focused on the role of TRPA1 in cold pain sensitivity in RA patients. In addition, it has been shown to play a role in mediating thermal (heat) and mechanical pain sensations in inflamed joints. One study showed that TRPA1 played a key role in both thermal and mechanical pain in CFA-induced arthritis (132). Similarly, application of the selective TRPA1 antagonist HC-030031 could significantly reduce mechanical hyperalgesia of the CFA-injected joint (108). Furthermore, intraarticular injection of CFA in TRPA1-/- mice inducing ipsilateral mechanical hyperalgesia was maintained for only 24 hours compared to 3 weeks in TRPA1 WT mice (133). These studies indicate that TRPA1 also takes part in inflammatory mechanical hyperalgesia and the transition from acute to chronic pain.

#### 5.2.3 The “love and kill” relationship between TRPV1 and TRPA1 in inflammatory pain

It is well manifested that TRPA1 is co-expressed in 60 – 75% of TRPV1-expressing sensory C-fiber nerves (107, 134). In addition, they interact with each other in pain perception at the central level. The frequency of spontaneous excitatory postsynaptic currents (EPSCs) in lamina I dorsal horn neurons were enhanced by CFA treatment, which could be attenuated with the employment of a specific TRPA1 or TRPV1 antagonist. TRPA1 mediated the increased presynaptic glutamate release to lamina I neurons and TRPV1 contributes to increased glutamatergic input in chronic inflammatory pain perception. Therefore, TRPA1 and TRPV1 cooperate at the spinal cord level, leading to the maintenance of chronic inflammatory pain (135).

Consistent with the above report on the involvement of TRPA1 in inflammatory mechanical hyperalgesia, in the CFA-induced inflammatory pain model, Tmem100 CKO mice showed attenuated mechanical hyperalgesia, indicating that TRPA1 participated in mechanical hyperalgesia. In DRG neurons, Tmem100 could be co-expressed with TRPA1 and TRPV1 and form a complex, which attenuated TRPV1’s inhibition of TRPA1, thereby selectively enhancing TRPA1 activities. Tmem100-3Q, a Tmem100 mutant had the opposite effect which enhanced the association of TRPA1 and TRPV1 and strongly inhibited the activity of TRPA1 in a TRPV1-dependent way. Tmem100-3Q CPP could mimic Tmem100-3Q’s effect and inhibits persistent pain by TRPA1. So, Tmem100-3Q CPP or molecules that have similar functions may be promising pain therapy for alleviating mechanical hyperalgesia (136). However, study also shown that reducing the link between TRPA1 and TRPV1 led to the reduction of TRPA1 function. They found that reduction of the A1-V1 complex significantly reduced TRPA1 sensitization and CFA-induced hypersensitivity (137). These findings suggest that there are certain functional molecules between TRPA1 and TRPV1 during they participate in the development and maintenance of hyperalgesia. Drugs or therapeutic strategies which are able to directly or indirectly target their interactions may shed new lights on potential analgesics and may have minor if any side effects.

### 5.3 Role of inflammatory mediators and neuropeptides in TRPs mediating inflammatory pain

Different pro-inflammatory cytokines are not only participants in inflammation, what’s more, each cytokine has its profile of effects on inflammatory pain. And most importantly, the formation of pain perception is determined by a variety of cytokines, and keep in mind that different stimuli and multiple pain receptors exist and work at the same time. Multiple sensory receptors, including TRPs, interact and influence each other to make certain pain perceptions. Due to the important role of TNF-α in RA, we will elaborate on the correlation between TNF-α and TRPs in mediating inflammatory pain. The influences of other inflammatory substances on TRPs in inflammatory pain perception are summarized in **Table 1**.

**Table 1:** Summary of RA related inflammatory stimuli on different subtypes of TRPs in inflammatory pain perception.

Subfamilies	Cytokines	Models of arthritis	Mechanisms of interaction between inflammatory stimuli and TRPs	nociceptive response	References
TRPV1	IL-1 ROS ROS	K/BxN serum transfer-induced arthritis AIA Carrageenan-induced arthritis CFA-induced hyperalgesia rats AIA rats	IL-1 receptor (IL-1R1) was highly expressed by a subpopulation of TRPV1+ dorsal root ganglion neurons. IL-1β up-regulated the expression of TRPV-1, and the expression of IL-1R1 in the lumbar DRGs was significantly increased. NADPH oxidase increased the production of ROS which promoted the level of PKC in DRG neurons, then activating the TRPV1. Activated Nox1/Nox4-ROS pathway sensitized TRPV1-dependent Ca^2+^ influx and CGRP releasing. FLS from AIA rats induced upregulation of pain-related receptors(neurokinin 1 receptor、TRPV1 receptor) in sensory neurons.	Increased mechanical allodynia Increased thermal hyperalgesia Increased thermal and mechanical hyperalgesia Increased mechanical hyperalgesia Increased thermal hyperalgesia	(138) (139) (140, 141) (142) (143)
TRPA1		RA patients	TRPA1 expression on peripheral blood leukocytes was increased.	Increased VAS pain scale	(144)
TRPC3	immune complex	Antigen-induced arthritis (AIA)	IgG-IC activated FcγRI which triggered TRPC channels through the Syk-PLC-IP_3_ pathway on DRG neurons.	Increased mechanical and thermal hyperalgesia	(145, 146)
TRPC5	Lysophosphatidylcholine (LPC)	CFA mice	The elevated concentrations of LPC-induced TRPC5-dependent neuronal activity in CFA-induced arthritis.	Increased mechanical hyperalgesia	(147)
TRPC5		CFA mice	The expression of TRPC5 was negatively correlated with the concentrations of inflammatory mediators and cytokines.	Decreased thermal and mechanical hyperalgesia	(148)
TRPV4	Protease-activated receptor-2 (PAR2)	Elastase stimulated mouse	Elastase induced a PAR (2)- and TRPV4-mediated influx of extracellular Ca^2+^ in nociceptors.	Increased mechanical hyperalgesia	(149)
TRPV4	IL-17A		IL-17A increased the expression of TRPV4.	Increased mechanical hyperalgesia	(150)
TRPV1 TRPA1	Oxidized phospholipids (OxPL)	CIA	OxPL evoked the CGRP release and activated TRPV1 and TRPA1.	Increased thermal and mechanical hyperalgesia	(151)
TRPV1 TRPC6	LPS	LPS-induced inflammatory	TRPV-1-mediated NLRP3 inflammasome activation. LPS-induced neutrophil and monocyte recruitment and promoted the activation of nociceptor TRPV1 and TRPA1.	Increased thermal hyperalgesia Increased mechanical and thermal hyperalgesia	(74, 152) (153)
TRPV1, P2X3R	Leukotrienes (LTs)	Mice pretreated with LTC4	Some types of LTs co-expressed in TRPV1 and P2X3-positive neurons.	Increased thermal hyperalgesia	(154)

#### 5.3.1 Differences between TRPA1 and TRPV1 in peripheral and central inflammatory perception of TNF-α induced inflammatory pain

TRPV1 are also downstream targets for various pro-inflammatory and pain-producing agents such as IL-1β and TNF-α. These compounds initiate an allosteric modification of the TRPV1 channel protein, resulting in an increase in the probability of channel opening or TRPV1 sensitized.

Paw withdrawal latency (PWL)was always taken as the thermal hyperalgesic threshold (155). TRPV1-/- mice had significantly higher baseline PWL than WT mice post-injection of TNF-a, the WT mice exhibited significantly reduced PWL compared to the TRPV1-/-mice, indicating that TNF-a induced thermal hyperalgesia was TRPV1 dependent. Moreover, contralateral uninjured hind paws also had thermal pain which was TRPV1-dependent, too. In addition to this, the local IL-1β generation in the contralateral paw, PKC and COX-2-derived prostaglandins were essential for the development of bilateral hyperalgesia (10). Researchers also concluded that most of the DRG neurons expressed the TNFR1 also expressed the TRPV1 receptor. TNF-α could up-regulate the expression of TRPV1 receptor, thus causing thermal hyperalgesia (156).

Some researchers completed their study which demonstrated that TNFα-mediated thermal hyperalgesia involves TRPV1 (10). They then wanted to investigate the role of TRPV1 and TRPA1 in the TNF-α induced mechanical hyperalgesia. By studying several models of inflammatory arthritis, they found that central distribution of TRPV1 was involved in TNF-α-induced mechanical hyperalgesia. The peripheral distribution of TRPA1 not only played in the occurrence of TNF-α induced mechanical hyperalgesia, but also act as a key role in the maintenance of CFA-induced mechanical hyperalgesia (133). In line with this result, researchers showed for the first time that TRPA1 has an important peripheral role in TNF-α-mediated mechanical hyperalgesia, which is different from TRPV1 distributed in the central nervous system in mechanical hyperalgesia (157). Thus, these studies present evidence that both TRPV1 and TRPA1 participate in TNF-α medicated inflammatory pain, but they may tend to have their separate strong points in peripheral and central inflammatory perception, which may direct us to explore the corresponding drug. Just as important, anti-TNF-α therapy has also been proven to have analgesic effects.

#### 5.3.2 TRPs collaborate with neuropeptides in perceiving inflammatory pain and promoting inflammation

Neuropeptide-expressing small-diameter sensory neurons are thought to be vital in inducing inflammatory hyperalgesic responses in inflammation. The CGRP peptide expression was increased after CFA injection compared to the control group and contributed to inflammatory hyperalgesia (158, 159). In arthritis mice, Concomitant with the disease progression, there was a significant increase in the density of CGRP+ nerve fibers in the synovium, as well as an enhancement in joint pain-related behaviors compared with the sham-injected mice (104, 160). CGRP-positive neurons partially overlapped with TRPV1 in DRG neurons. As a mutual, activated TRPV1 up-regulated CGRP expression, leading to the induction of inflammation pain (161). TRPV1 expression was also increased in small peptidergic (CGRP positive) neurons after injection of CFA and played a role in chronic thermal hyperalgesia and mechanical pain (126).

In addition to their role in pain perception, CGRP-secreting nerve fibers also contribute to RA inflammation. As highlighted and discussed at the beginning of this paper, CGRP is an extremely potent vasodilator, leading to edema formation and recruitment of inflammatory cells to the local sites. It also significantly elevated the production of inflammatory cytokines (such as IL-1β, IL-6 and TNF-α) in RA patients (162, 163). Neuropeptides may strongly enhance TRPV1 expression in RA synoviocytes. SP or CGRP in combination with TRPV1 could powerfully motivate the expression of pro-inflammatory cytokines IL-6 and IL-8 in RA synoviocytes, having no effects on healthy synoviocytes (164).

Neural TRPs have the potential role to orchestrate inflammatory signals with neuropeptides released by neurons within the synovial microenvironment of the RA-inflamed joints. Various inflammatory mediators and neuropeptides sensitize and regulate the activation threshold of TRPs, causing inflammatory pain. In turn, TRPs located on neurons not only sense pain, but also boost inflammation by promoting the production of pro-inflammatory neuropeptides. These findings help us understand the neuro-inflammation mutually influenced pain aggravation, as well as inflammation persistence. Moreover, it also proves that TRPs can be used as a new target for clinical treatment of RA.

## Conclusion

To sum up, evidence suggests the multiple functions of TRPs in RA pathogenesis, including joint swelling, neutrophil TEM and activation, angiogenesis and pain hyperalgesia. Ca^2+^ signals or potential induced by TRPs play a central role in transmitting inflammatory signals and amplifying inflammatory pain. TNF-α, VEGF and ROS are inflammatory molecules enriched in the joint microenvironment, acting on TRPs on ECs to increase the vascular permeability, which is the prerequisite for joint edema. TRPs-mediated Ca^2+^ influx into neutrophils plays an important role in their adhesion and TEM. Ca^2+^ signals caused by TRPs on neutrophils catalyze PADs activity and NETs formation, which enhancing the amount of auto-antigen in RA and leading to the formation of destructive autoantibodies ACPA. Though the relationship between TRPs and RA-FLS proliferation or apoptosis yet needs further investigation, TRPs can affect ECs migration and angiogenesis. Various inflammatory mediators and neuropeptides can sensitize and modulate the activation threshold of TRPs, resulting in the development of pain behaviors. TRPs can not only perceive cold and inflammatory stimuli but also communicate with pro-inflammation neuropeptides released by neurons within the RA-inflamed joints, resulting in persistent inflammation and pain hyperalgesia.

With scientists’ endeavors to explore TRPs over the years, many drugs targeting TRPs and antagonists interacting with inflammatory factors are in preclinical studies. Preclinical studies on TRPV1 as an analgesic have made fruitful progress. As TRPV1 agonists, capsaicin substances can cause TRPV1 desensitization of nerve endings when applied in a larger dose in the periphery, thus playing an analgesic role. Patches, creams, and sprays containing capsaicin have not only shown good analgesic effects in preclinical studies on neuralgia (165), joint pain (166), etc., but also have relatively few side effects. Antagonists targeting TRPV1 have significant analgesic effects, but blocking TRPV1 impairs the body’s perception of heat, leading to significant hyperthermia side effects (167). Therefore, the current direction of research is toward the development of antagonists that both reduce pain and less affect the body’s perception of temperature changes (168). At present, many synthetic antagonists for TRPA1 are gradually appearing, including Xanthine derivatives (HC-030031) and oxime derivative, which have been described in recent articles (169). From preclinical data, oxidative stress and activation of Schwann cell TRPA1 may serve as new targets for TRPA1 antagonists to relieve chronic pain in models of pain disorders. Studies shown that botulinum toxins (BoNTs) as a kind of neurotoxins had the ability to suppress the increase of neuropeptides (170). BoNTs acted as an analgesic by reducing TRPV1 expression (171). It not only presented analgesic effects in preclinical arthritis pain models, but also had very few side effects (172). Pre-clinical data on drugs derived from natural ingredients targeting TRPs have also made some progress. Tanshinone IIA (TIIA) is an important component of traditional Chinese medicine Danshen, which has achieved good curative effect in the treatment of nervous circulatory system. Studies have proved that it inhibited the release of inflammatory factors and reduced the expression of TRPV1 in the nervous system so as to reduce inflammation and anti-nociceptive pain in inflammatory arthritis (173). In addition, Paeoniflorin inhibited TRPV1 activation, thus exerting anti-inflammatory and analgesic effects without the hyperthermia side effects associated with traditional TRPV1 antagonists (174). S-(+)-dicentrine derived from camphor plants, reduced cold and mechanical pain in inflammatory mice by inhibiting TRPA1 activation (175). Not only has the anti-inflammatory and analgesic effect of traditional Chinese medicine (TCM), but other TCM methods have also shown a pleasing effect in relieving pain. The effect of electroacupuncture on pain relief has been widely recognized, but the specific mechanism is still unclear. Recent studies manifested that electroacupuncture reduced joint pain by interacting with TRPV1 (176). In addition, different acupuncture intensities and the distribution of nerve fibers where TRPV1 was located also vary the analgesic effect and the body parts relieved by electroacupuncture (177). Studies have shown inspiring efficacy in anti-inflammation and analgesic, although there are some side effects. This encourages us to continue to explore the mechanisms of TRPs about the unknown to better relieve the pain and suffering of RA patients.

## Author contributions

MN reviewed the literature and wrote the initial draft. MN, PL, and LB designed the article and critically revised the manuscript. FZ and RC collected the data. All authors have approved the manuscript and agreed to the published version of the manuscript.
